# Advanced Lasers and Their Applications in Dentistry

**DOI:** 10.3390/dj13010037

**Published:** 2025-01-16

**Authors:** Olivia Lili Zhang, Iris Xiaoxue Yin, Ollie Yiru Yu, Kenneth Luk, John Yun Niu, Chun Hung Chu

**Affiliations:** Faculty of Dentistry, The University of Hong Kong, Hong Kong 999077, China

**Keywords:** laser, dentistry, caries, laser fluorescence, photobiomodulation therapy, photodynamic therapy

## Abstract

The development of laser technology has revolutionized dentistry, offering complementary and alternative approaches to traditional techniques. Lasers have been successfully integrated into various dental procedures, enhancing treatment outcomes and patient care. Several types of lasers can increase the acid resistance of enamel, thus preventing caries. Laser fluorescence has been utilized for the pre-operative diagnosis of dental caries, enabling early detection and effective treatment planning. The therapeutic application of lasers in caries treatment aligns with the contemporary philosophy of minimally invasive procedures. Clinicians can use laser Doppler flowmetry as a supplementary tool for pulp vitality testing by detecting pulpal blood flow. Lasers are also employed in various pulp-related interventions, such as managing dentine hypersensitivity and performing root canal therapy. These procedures benefit from the precision and reduced invasiveness provided by laser technology. Furthermore, laser fluorescence serves as an additional tool for subgingival calculus detection. High-power and low-power lasers are used in both nonsurgical and surgical therapies to treat periodontal and peri-implant diseases, oral mucosa conditions, and even cancer based on their specific properties. Lasers are also utilized to accelerate bone regeneration, promote adhesive strength, and remove ceramic brackets. In summary, laser technology has significantly impacted contemporary dentistry by facilitating early diagnosis, minimally invasive treatments, and precise operative procedures, ultimately improving patient outcomes and expanding the scope of dental practice.

## 1. Introduction

The development of laser technology has revolutionized the management of oral disease. Oral diseases are still the most prevalent diseases globally, affecting over 3.5 billion people worldwide. The three common and significant oral diseases globally are dental caries, periodontal disease, and oral cancers [1]. Oral diseases are chronic and progressive in nature. For instance, dental caries is a lifelong condition that persists from early childhood through adolescence, adulthood, and later life [2]. Therefore, the management, including prevention, early diagnosis, and therapy of oral diseases, should be continuous. Lasers have been proven to be an effective tool not only for prevention but also for the early diagnosis and treatment of oral diseases [3]. First, lasers can be used for oral disease prevention [4]. In the prevention of dental caries, for example, inhibiting the biofilm and enhancing remineralization have been effective [5]. The widespread use of water fluoridation and fluoride-containing oral products has significantly reduced the prevalence and severity of dental caries. Topical fluoride application has also demonstrated efficacy. To improve topical fluoride efficiency, researchers have found that laser irradiation combined with topical fluoride treatment can induce an even higher increase in acid resistance to demineralization [6,7]. A ten-year literature review has shown that laser irradiation could be an alternative or synergistic approach to topical fluoridation for enamel caries prevention with longer-lasting effects [8].

Early and accurate detection of oral diseases is crucial. For instance, the detection of incipient caries might beneficially reduce the prevalence of caries and their socio-economic cost. X-ray is a common diagnostic tool. However, initial occlusal enamel caries are hard to be detected by radiography [9,10]. Laser fluorescence has been proposed as a diagnosis tool in the detection of caries lesions from various sites of the tooth for caries in both primary and permanent teeth [11,12]. Additionally, the laser device can monitor the caries process over time due to its good reproducibility. When it comes to laser treatment in dentistry, multiple studies have shown that laser is an effective tool for pre-operative and post-operative procedures. Biostimulation procedures can use photobiomodulation therapy [13], formerly known as low-level laser therapy, to activate regenerative and healing processes, reducing post-operative pain and inflammation [14]. Photodynamic therapy has been effectively utilized in the treatment of diverse pre-neoplastic and neoplastic diseases within the oral cavity [15]. This non-invasive approach can eliminate undesirable eukaryotic cells and pathogenic microorganisms [16]. Concurrently, high-power lasers have gained wide acceptance in therapeutic protocols [17]. Dental procedures that incorporate lasers tend to be less invasive, resulting in minimized post-operative pain. This can be especially beneficial for patients who suffer from dental anxiety or have a low pain tolerance. Additionally, the use of lasers can potentially reduce the necessity for local anesthesia and sutures, thereby enhancing patient comfort during the experience [18].

With advancements in laser technology and the availability of new wavelengths and modes, lasers can also be used to modify the surfaces of dental biomaterials such as implants, ceramics, and other materials used for restorative purposes [19]. Using lasers on these materials can improve their optical, tribological, biological, and other surface properties. For instance, implants modified using lasers demonstrate enhanced osseointegration and reduced chance of peri-implant inflammation [20]. Although lasers, which are versatile and precise instruments, have become increasingly integrated into dental care, the knowledge of clinical practitioners in this field is insufficient. The aim of this narrative review is to provide an overview of the role lasers play in contemporary dentistry, covering a broad range of clinical applications.

## 2. Application of Lasers for Caries Management

Dental caries refers to the localized destruction of dental hard tissues caused by acidic by-products from the bacterial fermentation of free sugars [21]. The caries process is dynamic, with alternating periods of demineralization and remineralization of the tooth structure related to fluctuations in the pH of the plaque biofilm [22]. Therefore, it is equally important to inhibit bacterial growth, avoid demineralization, and/or promote remineralization for caries management [23]. Laser technology has revolutionized the way dental professionals approach the prevention and treatment of dental caries or cavities. Lasers offer a minimally invasive and precise means of managing dental caries (Table 1).

Lasers can alter the composition or solubility of the dental substrate, which means they can be used to modify the structure of dental hard tissue to prevent dental caries. It is important to note that during the prevention procedure, the laser energy must be strongly absorbed and efficiently converted without damaging the underlying or surrounding tissues [24]. Carbon dioxide (CO_2_) lasers have a high absorption coefficient with hydroxyapatite. Several in vitro studies have been conducted to evaluate the prevention effect of CO_2_ lasers on enamel and dentine structures [25,26,27,28,29]. A literature review also confirmed that CO_2_ lasers are the most studied systems for caries prevention effects [8]. As for the mechanism, a literature review showed that CO_2_ laser with specific wavelengths alters the hydroxyapatite crystals of enamel, further reducing the acid reactivity of the mineral [24]. Another literature review showed that CO_2_ laser irradiation at 10,600 nm can reduce the carbonate content of the mineral and increase the microhardness of enamel [5].

Fluoride plays an important role in dental caries prevention [30]. For example, silver diamine fluoride is one of the topical fluoride application products. When applied to the tooth, it can kill bacteria and promote remineralization. The World Health Organization has recommended its use. However, the amount of topical fluoride taken up is usually limited to the surface layer [31]. Thus, the combined use of lasers and fluoride has been suggested to promote deeper fluoride penetration into tooth enamel and improve the efficiency of caries prevention. Studies have shown the combined effect of silver diamine fluoride and lasers in caries prevention [25,26]. Several in vitro studies demonstrated that CO_2_ laser of 9300 nm at 0.67 W with pulse mode on 0.8 mm^2^ spot area and 10,600 nm at 0.36 W with continuous wave mode on 0.019 cm^2^ area separately or combined use with silver diamine fluoride could enhance the resistance of enamel and dentine to cariogenic challenge [7,25,26]. CO_2_ laser irradiation and fluoride treatment showed a better effect on caries prevention than CO_2_ laser irradiation or fluoride alone. This combination allows for good prevention outcomes by using reduced irradiation energy density and fluoride levels, which enhances patient safety [24].

Erbium-doped yttrium aluminium garnet (Er:YAG) laser with a wavelength of 2940 nm is strongly absorbed by water. An in vitro study showed that the Er:YAG laser significantly prevents enamel demineralization [32]. The combination use of Er:YAG laser with silver diamine fluoride has been investigated and showed that silver diamine fluoride application followed by Er:YAG laser irradiation on a dentine surface increased its resistance to cariogenic biofilm challenge [33]. Another in vitro study showed that the combined use of a diode laser at 445 nm with 0.3 W, continuous wave mode on 0.2 cm^2^ area for 60 s, and silver diamine fluoride has a superior effect on preventing enamel demineralization and inhibiting cariogenic bacteria [6]. Different laser systems have been conducted and showed a positive effect of varied degrees on caries prevention [34]. A study found that the Er:YAG laser can safely increase the acid resistance of the dentine surface of the root. However, a study reported no synergistic effect when combining laser irradiation with fluoride [28]. Therefore, further studies are necessary to clarify the prevention mechanisms of dental lasers on enamel and dentine caries and the combined effect with or without fluoride.

Caries detection and early diagnosis are crucial for dental care. It is essential to identify dental caries early to prevent them from leading to more serious oral health issues. Early and accurate detection of incipient caries can help reduce socio-economic costs and support the principle of minimally invasive dentistry. One of the most promising techniques for caries detection is laser-induced fluorescence [35]. A diode laser with a wavelength of 655 nm (DIAGNOdent and DIAGNOdent-Pen) has been used to detect caries.

Laser-induced fluorescence is a non-invasive technique that uses a low-level laser to illuminate the teeth and detect fluorescence emissions that indicate the presence of dental caries. When the laser is directed at the teeth, it excites the molecules in the tooth structure, causing them to emit a fluorescent light. The fluorescence emissions are then analyzed to determine the presence and severity of caries [35]. Unlike traditional caries detection methods, such as X-rays, laser-induced fluorescence does not require the use of radiation. Additionally, laser-induced fluorescence is a highly accurate technique that can identify early-stage caries and hidden caries that may not be visible to the naked eye. Laser fluorescence detection devices can provide specific information like the location and extent of caries [10,36]. However, laser-induced fluorescence is also prone to false-positive diagnoses, which may lead to overtreatment. Therefore, laser-induced fluorescence is recommended as a supplementary diagnostic tool rather than a primary diagnostic tool for caries detection [11]. A near-infrared light transillumination camera system (DIAGNOcam) using a laser wavelength of 780 nm has been introduced to the dental market. Cracklines, defective occlusal margin, and early proximal demineralization can be easily detected.

In addition to detecting early caries using laser fluorescence, another fluorescence-aided caries excavation method with a violet laser with a wavelength of 405 nm (SIROInspect) is used to distinguish infected and non-infected dentine further to avoid over-excavation without removing the healthy dentine [37]. When the tooth is exposed to violet light, the infected dentine emits red fluorescence [38,39]. An in vitro study demonstrated that the fluorescence-aided caries excavation method is an appropriate caries-detecting system without affecting the pulp chamber’s health and can be safely used in the primary teeth [38].

In the treatment of caries, the application of lasers aligns with the modern concept of comfort dentistry and minimally invasive procedures. Compared to traditional instruments used in caries treatment, lasers offer a relatively more comfortable approach [40]. Clinical practitioners acknowledge that high-speed rotating instruments often generate vibrations in the teeth, noise, and discomfort, which can cause dental fear, particularly in children. The use of lasers within the oral cavity is comparatively safer as it does not involve any rotating or cutting tools.

Lasers induce fewer vibrations in the tooth, and low-pitch noise is less than high-speed turbines. Furthermore, laser treatment is a painless process [41]. Even in cases requiring local anesthesia, prior laser irradiation on the mucosa can enhance the depth of anesthesia penetration within the mucosal layers, thereby improving the analgesic effect [42]. A systematic review demonstrated that most children preferred the Er:YAG laser due to its ability to reduce pain and enhance overall comfort [43].

The principle of minimally invasive caries treatment necessitates the removal of only those dental structures that are irreversibly decayed and infected with bacteria [44]. The Erbium family of lasers, which includes Er:YAG and Er,Cr:YSGG (wavelength 2780 nm), with their high affinity for water, can be used for the ablation of dental hard tissues. Water molecules absorb laser energy and undergo micro-explosion, generating high pressure, which results in the removal of tooth structure [45]. Because carious tissue contains a higher water content than healthy tissue [46], at the ablation threshold below healthy tissue, the Erbium family of lasers can selectively remove only the decayed tissue, leaving the healthy tissue untouched. This helps to preserve the structural integrity of the tooth and minimizes the need for extensive restorative treatment.

Lasers also decontaminate the affected area, thus reducing the risk of further infection and secondary caries. The Erbium family of lasers has been approved for caries removal and cavity preparation [43]. Moreover, because Erbium family lasers can work on both soft and hard tissue, they can be used to expose subgingival cavity margins, which will be beneficial for cavity preparation. The 9300 nm (Solea) and 9600 nm CO_2_ wavelengths are most highly absorbed by hydroxyapatite, which is the main component in dental hard tissue. The 9300 nm short-pulsed CO_2_ laser is currently in the dental market but not distributed globally. It has been shown to provide a smooth cavity margin with effective ablation efficiency [47].

In addition to caries removal, laser treatment may also enhance the adhesion of restorations [48,49]. This can be achieved in two ways. Firstly, laser preparation can increase the surface area for bonding. An in vitro study demonstrated that using the Er:YAG laser could open dentinal tubules without forming a smear layer, which can improve the bonding strength between dentine and composites [48]. Researchers have also explored alternative protocols, such as modifying parameters or incorporating additional procedures, to achieve better resin–dentine bonding [49]. For instance, a Q-switched Er:YAG laser with a shorter pulse-treated dentine has a higher bonding capacity compared to a traditional Er:YAG laser [40]. Another study indicated that supplementary low-power (30 mJ) Er:YAG laser irradiation enhances resin–dentine bonding [49].

The durability of the resin–dentine interface is often questioned due to the complex oral biofilms that accumulate acid-producing bacteria along the interface, leading to failure [50]. Therefore, the development of a dentine adhesive with antibacterial properties is crucial for successful tooth bonding. Antimicrobial photodynamic therapy applied to photosensitizer-doped dentine adhesive materials has demonstrated increased bond strength and superior antimicrobial capability [51].

The mechanism behind antimicrobial photodynamic therapy involves the generation of reactive oxygen species through the photosensitizer upon irradiation. Reactive oxygen species can disrupt bacterial growth and replication by causing DNA damage and interfering with cellular metabolism. For instance, a study used riboflavin as the photosensitizer in dentine adhesive and found that riboflavin photodynamic therapy inhibited bacterial growth and increased bond strength [50]. Overall, dental lasers are safe and effective tools for managing dental caries. They enable dental professionals to provide a preventive, minimally invasive, and comfortable caries treatment approach.

## 3. Application of Lasers for Pulp-Involved Disease Management

The progression of caries into deep dentine ultimately results in inflammation of the pulp, followed by pulp necrosis and, finally, apical periodontitis if adequate restorative treatment is not undertaken. Furthermore, chronic stimuli, injuries, and other conditions can lead to dentinal hypersensitivity, pulp necrosis, and root canal obliteration. Maintaining pulp vitality, preventing the spread of infection, and developing biologically minimal-invasive therapies are essential themes within contemporary clinical endodontics [52]. The use of lasers in this field is in line with these themes (Table 2).

Accurate diagnosis of pulp disease is directly related to the choice of treatment plan and affects the prognosis of the disease. Optimal diagnostic techniques should accurately describe the continuum of pulpal inflammation and expect the healing potential of the affected tissue based on the inflammatory status [53]. The key point is to assess the pulpal vitality of individual teeth. Particularly for children and young patients, accurate detection of pulp vitality is crucial to preserve dental pulp functionality and promote tooth root development. Thermal and electric pulp testing remain the appropriate clinical tests to use. However, both thermal and electric pulp testing are crude and non-quantitative [53]. These diagnostic methods are subjective because they do not directly determine pulp vitality but rather infer it through patients’ sensitivity to stimuli [54,55]. Direct vitality tests should indicate the presence or absence of blood flow [54]. Laser Doppler flowmetry is a true pulp vitality test because it can detect pulpal blood flow without relying on the patient’s response and is thought to provide more accurate pulp status [55]. This is a non-invasive, objective, painless, and semi-quantitative measurement.

Laser Doppler flowmetry has been verified to provide reliable and reproducible results [56]. A systematic review showed that laser Doppler flowmetry is one of the most accurate diagnostic methods [55]. An in vivo study assessed the efficacy of the laser Doppler flowmetry technique in diagnosing pulp vitality after dental trauma and showed that laser Doppler flowmetry is a useful tool for diagnosing traumatic pulp necrosis [56]. However, laser Doppler flowmetry has some drawbacks. Changing the probe position on the tooth might affect the output signals because this device is sensitive [57]. Thus, it is important to ensure the reflected signal only comes from the pulp during practice. Additionally, the incident light must reach moving blood cells through the mineral tissues, which may limit the penetration of the laser beam into the tooth [58]. Therefore, laser Doppler flowmetry can be used as a supplementary diagnostic tool but not to replace radiological or clinical examinations.

For the treatment aspects, lasers can be used alone or in combination with classic desensitizing interventions to manage dentinal hypersensitivity [59]. Dentinal hypersensitivity is due to the exposure of open dentinal tubules. Traditional therapy includes two aspects: one is occluding dentinal tubules, and the other is blocking nerve activity. Lasers can promote dentinal tubule occlusion through the alteration of the surface or reduce nerve sensitivity by protein coagulation. Diode lasers with different wavelengths, Erbium family lasers, neodymium-doped yttrium aluminum garnet (Nd:YAG) laser (wavelength of 1064 nm), and CO_2_ lasers (wavelength of 9300 nm, 9600 nm, and 10,600 nm) have been employed to relieve the pain of dentinal hypersensitivity [60]. A literature review showed diode, Nd:YAG, Er:YAG, and CO_2_ lasers cause desensitization due to dentinal tubule occlusion [61]. An in vivo study found that a diode laser at 980 nm with a delivery output of 1 W coupled to graphite paste is considered to be effective in treating dentinal hypersensitivity without any injury to the dental pulp [62]. Pandey R et al. found that diode laser at 810 nm with 0.5 W can reduce dentinal hypersensitivity [63]. Umana M et al. concluded that diode lasers at 0.8 W and 1 W for 10 s in continuous mode can effectively treat dentinal hypersensitivity without damaging dental pulp [64]. Nd:YAG laser was reported to be the most effective laser [65]. Nd:YAG laser of 1064 nm wavelength at 1 W had a good ability to occlude dentinal tubules and reduce the diameters of the tubules [65]. A study found that Nd:YAG laser at 0.5 W effectively manages dentinal hypersensitivity [66].

A systematic meta-analysis concluded that laser application may improve pain intensity. However, because of the heterogeneous included studies, long-term, well-designed, randomized controlled trials are required to evaluate the effectiveness and safety of the treatment of dentinal hypersensitivity [60].

Furthermore, lasers can be used for vital and non-vital pulp therapies. Vital pulp therapy aims to treat reversible pulpal inflammation, particularly for primary teeth and immature permanent teeth. It includes three therapeutic approaches: indirect pulp therapy, direct pulp capping, and pulpotomy [67].

For vital pulp, lasers have the ability to decontaminate and vaporize tissue and coagulate and seal small blood vessels in reversible hyperemia [12]. Erbium family lasers, diode lasers, and CO_2_ lasers have been used to treat vital pulp [59]. Different parameters are set according to various treatment [59]. Diode laser at 1.5 W with continuous wave mode, Er,Cr,YSGG laser at 0.5 W with non-contact mode for 10 s, and CO_2_ laser at 0.5 W with repeat mode for 15 s have been used for pulp capping [59]. 

Non-vital pulp therapy is a root canal treatment that includes disinfection of the root canal system, root canal shaping, root canal irrigation, removing the smear layer on root canal dentine walls, and root canal obturation. CO_2_ lasers, Erbium family lasers, Nd:YAG laser, Nd:YAP laser (wavelength 1340 nm), and diode lasers have been applied and used in different procedures [59].

Lasers can also be applied to alleviate pre-operative pain and post-operative pain in endodontic treatment [59]. Various laser therapies in root canal disinfection have demonstrated that the combination of antimicrobial photodynamic therapy with common antimicrobial irrigants can provide a synergetic effect and can be considered as an alternative to conventional disinfection methods for persistent infections [68]. However, the optimal efficacy of laser therapy applications in root canal disinfection with or without various irrigants still remains unattainable [68]. More standardized protocols are needed to optimize the potential outcomes.

## 4. Application of Lasers for Periodontal and Peri-Implant Disease Management

Periodontal and peri-implant diseases are plaque-associated pathological conditions that affect the tissues and structures surrounding and supporting the teeth or implants. Periodontal disease impacts the gums, namely gingivitis, and causes the subsequent progressive loss of the periodontal ligament and alveolar bone, known as periodontitis [69]. Meanwhile, peri-implant disease affects the peri-implant mucosa and leads to the progressive loss of supporting bone, referred to as peri-implantitis [70]. Anti-infective treatment strategies should be employed to manage these types of diseases. Lasers can be used in periodontal diagnosis and treatment through non-surgical and surgical approaches [71]. Table 3 shows the application of lasers for periodontal and peri-implant disease management.

Subgingival scaling and root planning remain the most effective approaches to eliminating the source of infection for periodontal treatment. The aim of subgingival scaling and root planning is the complete removal of subgingival calculus. Tactile examination using a periodontal probe can be used to evaluate the effect of calculus removal [72]. However, the use of a probe has been reported to yield false negatives [72]. An alternative approach to detecting subgingival calculus involves using laser fluorescence. Based on the phenomenon that subgingival calculus exhibits autofluorescence when exposed to 655 nm laser light [73], this laser has been commonly used for identifying subgingival calculus. In vitro and in vivo studies have been conducted and provided positive results [72,73]. Therefore, it may be used as an additional tool for calculus detection in non-surgical periodontal surgery [74].

The high-power lasers can be used as monotherapy or as an adjunct therapy to the subgingival scaling and root planning mechanical treatment. In order to strengthen the effects of subgingival scaling and root planning, high-power lasers such as the Erbium family laser, Nd:YAG laser, and diode laser are indicated to remove dental calculus and sulcular epithelium, as well as to promote the reduction of bacteria in the periodontal pocket [71,75]. 

A diode laser at wavelength 808 nm with 1 W used as monotherapy alongside subgingival scaling and root planning showed significant effectiveness [71]. However, Nd:YAG laser at 400 mJ/pulse and diode laser at wavelength 940 nm with 0.8 W demonstrated no additional clinical benefits compared to subgingival scaling and root planning [71]. It has been noted that the severity of periodontitis can impact the clinical outcomes of laser treatment [71]. Additionally, a potential concern is that the thermal effects of high-power lasers may cause damage to pulp tissue [76].

On the other hand, photobiomodulation therapy, formerly termed low-level laser therapy, mainly targeting soft tissue, is recommended for its photochemical role in anti-inflammation, biostimulation, and analgesia. It can be used as an adjunct method to non-surgical therapy for periodontal disease. 

Diode lasers with wavelengths in the red and near-infrared range and potassium titanyl phosphate (KTP) lasers are commonly used for low-level laser therapy. A meta-analysis of clinical studies has indicated that photobiomodulation therapy has short-term additional benefits when combined with subgingival scaling and root planning [76]. Diode laser at continuous mode with wavelength from 630 nm to 830 nm and energy dosage ranging from 0.12 to 12 J per tooth was used in most studies [76]. However, this study was based on a limited number of included studies. Thus, long-term follow-up studies using standardized laser parameters are required.

Antimicrobial photodynamic therapy has also been used to treat periodontal disease. It produces reactive oxygen species, which target and damage bacterial cells. Antimicrobial photodynamic therapy has been clinically evaluated as adjunctive therapy for the treatment of periodontal diseases and implant decontamination [15]. 

In addition, non-surgical periodontal treatment combined with lasers can be used for subjects with periodontitis and type 2 diabetes mellitus [77]. However, some studies have shown controversial results on antimicrobial photodynamic therapy in periodontal diseases [71]. A meta-analysis showed that available evidence on adjunctive therapy with lasers and antimicrobial photodynamic therapy is limited [78]. Thus, well-designed studies are needed to further evaluate the effectiveness of antimicrobial photodynamic therapy.

For surgical therapy, lasers at high power can be used in gingivectomy, gingivoplasty, second-stage surgery of submerged implants, ulectomy or ulotomy, flap de-epithelialization in regenerative procedure, osteotomy and osteoplasty [71]. CO_2_ lasers at a wavelength of 10,600 nm, diode lasers with wavelengths of 810 nm to 1064 nm, Erbium family lasers, Nd:YAG lasers, and Nd:YAP lasers are indicated to have the ability to perform these surgeries. Er:YAG laser at 30 Hz and 80 mJ/pulse could effectively and safely remove granulation tissue [75]. The mechanism is that when these lasers interact with the tissue, the laser energy absorbed by the target chromophore produces a photothermal effect on the target that generates coagulation, incision, and vaporization of the soft tissues.

Low-level lasers can also be applied as an adjunct to operative procedures. Using low-level lasers such as antimicrobial photodynamic therapy can promote the repair of gingival and mucous tissues, accelerate bone tissue repair, and reduce post-operative symptoms of periodontal surgery. A systematic review demonstrated that although antimicrobial photodynamic therapy can provide improvements, these additional gains did not lead to significant benefits over conventional periodontal therapy [79]. Hence, similar to non-surgical laser applications, more evidence-based protocols are required for employing lasers for managing periodontal diseases [15].

## 5. Application of Lasers for Oral Mucosal Disease and Oral Cancer Management

Multiple pathological disorders of varying etiology and severity can affect the oral mucosa tissues of the oral cavity, including the gums, cheeks, lips, and tongue. Oral mucosa diseases are divided into the following categories: ulcerative, vesiculobullous, white and red, pigmented, and papillary or hyperplastic. Common oral mucosa diseases include oral lichen planus, recurrent aphthous stomatitis, and oral leukoplakia. Some oral mucosa diseases are considered premalignant conditions that can increase the risk of developing oral cancer. Therefore, early diagnosis and treatment of oral mucosa diseases are essential to prevent complications and improve the quality of life. Table 4 shows the application of lasers for oral mucosal disease and oral cancer management.

As described earlier in surgical therapy for periodontal disease, various types of lasers have been widely used in the removal of oral soft tissue lesions. High-power lasers, including CO_2_ laser, diode laser, Erbium family laser, Nd:YAG laser, and Nd:YAP laser, used as incision instruments, have been shown to enhance and improve clinical and surgical procedures. The basis of laser tissue incision is the photothermal effect. However, thermal damage needs to be considered. A recent in vitro study showed that a diode laser at 810 nm has better coagulation for highly vascularized tissue, while a diode laser at 980 nm provides better ablation. Therefore, the dual-wavelength diode laser (810 nm and 980 nm) improves coagulation and ablation with minimal thermal damage [80]. The Er,Cr:YSGG laser with 2.5 W output power and 50 mJ pulse energy can be used more effectively and safely for oral soft tissue incision than the dual-wavelength diode laser [80].

Low-power lasers have also been widely applied for the treatment of oral mucosa diseases. Photobiomodulation therapy can modulate the proliferation of fibroblasts, stimulate the differentiation of epithelial cells, and inhibit several nociceptive stimuli. All of these are essential for wound healing, tissue regeneration, and pain reduction.

A literature review showed that photobiomodulation therapy presents as a reasonable treatment modality both in recurrent aphthous stomatitis and oral lichen planus and can be incorporated into standard treatment [81]. The suggested parameter settings for a diode laser are a wavelength between 670 and 810 nm, power output ranging from 40 mW to 0.5 W, and exposure time between 40 s and 180 s [81]. A systematic review suggested that low-level laser therapy may be an effective alternative in the treatment of burning mouth syndrome [82]. However, the diversity of photobiomodulation therapy parameters used in the included studies impedes the unambiguous interpretation of the results. Hence, further well-conducted and standardized studies are required to define the efficacy of photobiomodulation therapy for oral mucosa diseases.

In addition, photobiomodulation therapy is suggested for the prevention of oral mucositis in specific cancer patient populations who are undergoing high-dose chemotherapy, radiation therapy, and hematopoietic stem cell transplantation [83]. It is an effective approach to reducing oral mucositis symptoms, including duration, severity, and pain. A systematic review conducted by the Mucositis Study Group of the Multinational Association of Supportive Care in Cancer/International Society for Oral Oncology provided support for the use of specific settings of photobiomodulation therapy for the prevention of oral mucositis [84]. Recommended intra-oral photobiomodulation therapy protocols for the prevention of oral mucositis have been shown in these guidelines [84].

In addition to photobiomodulation therapy, antimicrobial photodynamic therapy is another potential alternative treatment for oral mucosa diseases. Antimicrobial photodynamic therapy has shown improvements in treating oral lichen planus. Another literature review cautiously concluded that antimicrobial photodynamic therapy may be a potentially effective and safe treatment for oral mucosa infections. However, the dose of photosensitizer, strain of microbes, and irradiation parameters may affect the results of antimicrobial photodynamic therapy [85]. Further studies, including standardized methods of measurement and long-term randomized clinical trials, are required to optimize the most efficient type and dose of photosensitizer, as well as the radiation parameters.

Even though the effectiveness of antimicrobial photodynamic therapy is questionable in the treatment of oral soft tissue diseases, antimicrobial photodynamic therapy offers another option for oral malignant and premalignant lesions [86]. It has been regarded as the fourth modality, with the first three being surgery, radiotherapy, and chemotherapy [87]. It can be used as a standalone intervention or as an adjunct to other modalities. It is a minimally invasive surgical tool. It can be applied repeatedly at the same site with no cumulative toxicity. In the treatment procedure, the photosensitizer is selectively retained in the target tissue, and then the laser light can activate the drug and initiate the photochemical reaction.

A review assessed the effect of photodynamic therapy on oral squamous carcinoma in vitro studies and indicated the efficacy of photodynamic therapy against squamous carcinoma, especially for the initial clinical stages of cancer [88]. An in vitro study found that photodynamic therapy is efficient in early squamous carcinoma and oral leukoplakia, classified as oral precancerous lesions. The concentration of photosensitizer is related to the distribution of epithelial cells and mesenchymal stem cells in oral leukoplakia and squamous carcinoma [89].

## 6. Application of Lasers for Orthodontics

Orthodontic treatment involves tooth movements relating to interactions of teeth with their supportive periodontal tissues. Orthodontic–periodontal problems may be caused during the procedures [90]. Lasers have been widely used in the treatment of periodontal diseases, which have been described in the periodontal section of this manuscript. Researchers also focus on assessing the effect of photobiomodulation therapy on orthodontic dental alignment based on the ability of photobiomodulation therapy to accelerate the remodeling of alveolar bone.

A systematic review found that photobiomodulation therapy can be used as a complement to rapid maxillary expansion during orthopedic and orthodontic treatments. It can accelerate bone regeneration and reduce the time of consolidation of the maxillary [91]. Photobiomodulation therapy may have a positive effect on accelerating dental alignment. However, more high-quality studies are necessary to better understand this effect due to the variations of studies in this field [92].

Increasing the number of *Streptococcus mutans* during orthodontic therapy may cause dental caries. An in vitro study evaluated the number change of *Streptococcus mutans* around orthodontic brackets using antimicrobial photodynamic therapy with a blue diode laser (wavelength 445 nm) [93]. The results suggested that antimicrobial photodynamic therapy using curcumin as a photosensitizer can effectively reduce the *Streptococcus mutans* around the brackets. Therefore, antimicrobial photodynamic therapy can be used for caries prevention during orthodontic therapy. Table 5 shows the applications of lasers for orthodontics.

High-power laser applications in orthodontic treatment mainly focus on promoting adhesive strength and removing ceramic brackets [94]. A preliminary study demonstrated that Er:YAG laser increases enamel roughness in contrast to 37% orthophosphoric acid etching, which can improve the bond strength of orthodontic brackets to the enamel surface [95]. Er:YAG laser parameters were as follows: energy: 100 mJ, frequency: 10 Hz, exposure time: 10 s [95]. Er,Cr:YSGG laser has also been assessed for efficiency on shear bond strength and found that Er,Cr:YSGG at 2 and 2.5 W for 10 s has a higher bond strength compared to acid etching [96]. The residual oxygen after bleaching may interfere with the adhesion of brackets to the enamel surface.

An in vitro study evaluated the influence of Er:YAG laser as an etching technique on the bond strength of orthodontic brackets to the enamel surface after bleaching [97]. The results showed that the Er:YAG laser increases the bond strength of brackets to the enamel surface after bleaching. In this case, bonding the brackets may be performed on the same day after bleaching.

On the other hand, for the debonding effect, researchers refer to the degradation of the bonding resin using lasers as photoablation [98]. A systematic review evaluated the Er:YAG laser debonding effect and found that Er:YAG laser debonding could reduce the risk of enamel damage (fracture or cracks) and low thermal exhaustion in relation to the pulp but increased enamel surface roughness [99]. However, the damage to dental pulp and enamel surface caused by laser debonding is still inconclusive [100].

## 7. Clinical Application and Protocol of Lasers in Dentistry

**Figure 1:**
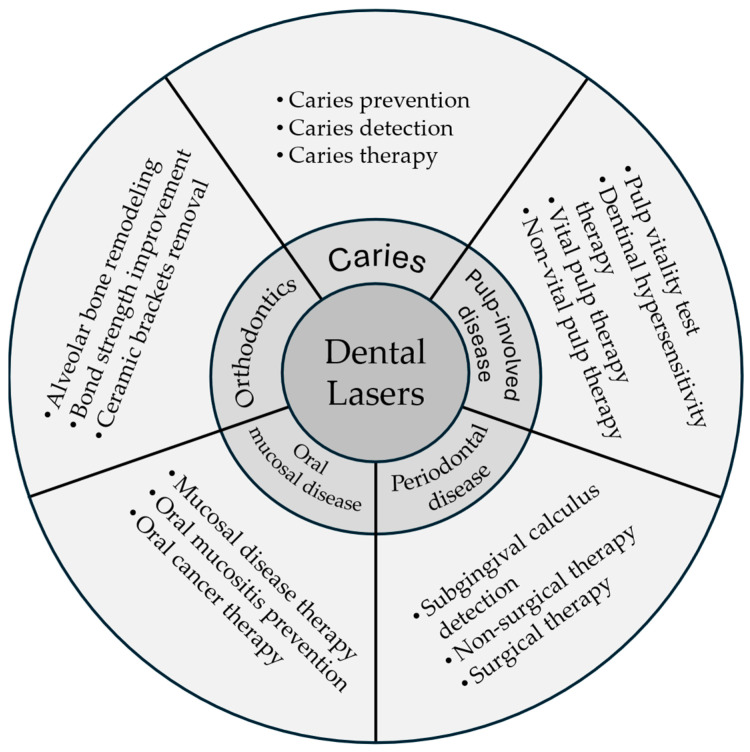
Lasers applications in dentistry.

The development of laser technology has revolutionized dentistry, offering complementary and alternative approaches to traditional techniques. Lasers have been successfully integrated into various dental procedures, enhancing treatment outcomes and patient care. Several types of lasers can increase the acid resistance of enamel, thus preventing caries. This narrative review provides an overview of the role lasers play in contemporary dentistry, covering a broad range of clinical applications (Figure 1). The purpose is to promote knowledge exchange in laser dentistry, benefiting patients and dental professionals. Table 6 is a suggested clinical protocol for applying lasers in dental treatment, covering patient assessment, equipment setup, procedure execution, and post-procedure care. By following a well-prepared protocol, clinicians can effectively integrate laser treatment into their practice to enhance the quality of care and patient outcomes.

## 8. Limitations and Challenges

While laser technology offers numerous benefits in clinical practice, there are notable issues and challenges associated with its adoption. Establishing and maintaining a safe environment for patients and staff at all times is a constant goal. To properly manage laser safety, it is important to have knowledge of laser standards, identify hazards and risks, implement appropriate control measures, and conduct consistent program audits to demonstrate quality assurance [101].

Clinicians and assistants need to have a solid understanding of laser science. Clinicians should comprehend the types of lasers, their applications, and safety protocols to competently and safely use laser technology. Incorrect settings or improper use can result in erythema, skin hyperpigmentation, thermal injury, and eye injury. Certain laser treatments may cause pain or discomfort during and after the procedure. Specific education and training are necessary for different wavelengths, systems, delivery devices, and applications due to hazard variations [101].

Assessment of risk level is also crucial. The risk level may vary with each laser system, depending on the delivery device, power parameters, target tissues, and operators’ experience. Eye protection is essential when exposed to the laser environment, as damage to various eye structures depends on the laser’s wavelength [101].

Clinicians often face challenges in delivering laser treatments because mastering laser technology requires significant time and effort to become proficient. Integrating laser technology into existing conventional practice workflows can be complex and disruptive. More clinical studies are needed to confirm the ideal laser parameters for specific clinical purposes and validate their effectiveness. Moreover, the higher costs associated with laser procedures may not be covered by insurance, resulting in higher out-of-pocket expenses for patients.

## 9. Conclusions

Dental laser technology provides alternative or supplementary treatments for various oral diseases. It can be utilized for caries detection, prevention, and restoration, as well as for pulp-involved diseases such as pulp vitality testing, dentinal hypersensitivity treatment, and vital or non-vital pulp therapies. In cases of periodontal or peri-implant diseases, lasers can detect subgingival calculus and be used alone or in combination with conventional treatments. Additionally, lasers can help prevent oral mucosal diseases in specific cancer patient populations and can be employed for surgical treatment of common mucosal diseases, premalignant lesions, and oral malignancies. Orthodontic therapy can also benefit from dental laser technology, including soft tissue surgery, accelerating dental alignment, and improving bracket bonding strength. Laser therapy offers a precise, gentle, and efficient approach that can enhance outcomes and minimize patient discomfort. However, standardized protocols for laser application in various oral diseases are still limited, and further research is needed to better understand tissue response to laser therapy and to establish standardized protocols for using lasers in different oral diseases.

## Figures and Tables

**Table 1:** Application of lasers for caries management.

Laser Application	Irradiation Effects	CO_2_ Laser	Diode Laser	Er,Cr:YSGG Laser	Er:YAG Laser	Nd:YAG Laser
Caries prevention	Modify dental hard tissue Enhance acid resistance Improve fluoride uptake	Yes	Yes	Yes	Yes	Yes
Caries Detection	Detect dental caries		Yes			
Caries treatment	Remove carious lesions Prepare cavity Enhance bonding	Yes		Yes	Yes	

**Table 2:** Application of lasers for pulp-involved disease management.

Laser Application	Irradiation Effects	CO_2_ Laser	Diode Laser	Er,Cr:YSGG Laser	Er:YAG Laser	Nd:YAG Laser
Pulp vitality test	Detect pulpal blood flow		Yes			
Dentin hypersensitivity therapy	Promote dentinal tubule occlusion Reduce nerve sensitivity	Yes	Yes	Yes	Yes	Yes
Vital pulp therapy	Decontaminate affected tissue Vaporize tissue	Yes	Yes	Yes	Yes	
Non-vital pulp therapy	Decontaminate root canal Shape root canal Irrigate root canal Remove smear layer		Yes	Yes	Yes	Yes

**Table 3:** Application of lasers for periodontal and peri-implant disease management.

Laser Application	Irradiation Effects	CO_2_ Laser	Diode Laser	Er,Cr:YSGG Laser	Er:YAG Laser	Nd:YAG Laser
Subgingival calculus detection	Identify subgingival calculus		Yes			
Non-surgical therapy	Remove calculus Remove sulcular epithelium Reduce bacteria (antimicrobial photodynamic therapy) Anti-inflammation (photobiomodulation therapy) Release pain		Yes	Yes	Yes	Yes
Surgical therapy	Remove sulcular epithelium Incise gingival Ablate bone tissue Promote tissue healing	Yes	Yes	Yes	Yes	Yes

**Table 4:** Application of lasers for oral mucosal disease and oral cancer management.

Laser Application	Irradiation Effects	CO_2_ Laser	Diode Laser	Er,Cr:YSGG Laser	Er:YAG Laser	Nd:YAG Laser
Mucosal disease therapy	Inhibit bacteria (antimicrobial photodynamic therapy) Remove mucosal lesion Promote tissue healing (photobiomodulation therapy) Reduce pain (photobiomodulation therapy)	Yes	Yes	Yes	Yes	Yes
Oral mucositis prevention	Anti-inflammation (photobiomodulation therapy)		Yes			
Oral cancer therapy	Minimal-invasive surgical therapy (photodynamic therapy)		Yes			

**Table 5:** Application of lasers for orthodontics.

Laser Application	Irradiation Effects	CO_2_ Laser	Diode Laser	Er,Cr:YSGG Laser	Er:YAG Laser	Nd:YAG Laser
Alveolar bone remodeling	Alveolar bone remodeling		Yes			
Bond strength improvement	Bond strength improvement			Yes	Yes	
Ceramic brackets removal	Ceramic brackets removal	Yes	Yes		Yes	Yes

**Table 6:** Clinical protocol for laser treatment in dental practice.

Protocol	Item	Description
Assessment and Selection	History Taking	Review the patient’s medical and dental history
	Initial Consultation	Conduct a thorough examination to assess suitability for laser treatment
	Informed Consent	Explain the laser procedure, benefits, risks, and alternative treatments
Pre-Procedure Preparation	Equipment Selection	Choose the appropriate type of laser based on the specific dental procedure
	Area safety	Implement safety protocols like using warning signs and securing the treatment area
	Personnel Safety	Ensure all personnel and the patient wear appropriate protective eyewear
Equipment Setup	Calibration	Calibrate the laser device according to the manufacturer’s instructions
	Testing	Perform a test fire to ensure the laser is functioning correctly
	Sterilization	Sterilize all laser handpieces and accessories to prevent cross-contamination
Procedure Execution	Anesthesia	Administer local anesthesia if required for patient comfort
	Laser Settings	Adjust the laser settings, including power, pulse duration, and frequency
	Mode selection	Use appropriate laser mode—continuous or pulsed mode
	Technique	Maintain the correct distance and angle between the laser tip and the tissue
	Cooling	Ensure tissue cooling to prevent thermal damage
	Monitoring	Monitor the patient and tissue response during the procedure
Post-Procedure Care	Immediate Care	Assess signs of complications, such as bleeding or tissue damage
	Patient instructions	Provide instructions like pain management, oral hygiene, and activity restrictions
	Follow-Up	Schedule follow-up appointments to monitor healing and treatment success
Documentation and Reporting	Record Keeping	Document procedure, laser settings, treatment details, and adverse events
	Evaluation	Evaluate the treatment outcomes and patient satisfaction

## Data Availability

The original contributions presented in the study are included in the article material; further inquiries can be directed to the corresponding author.
